# Ozena

**Published:** 1909-10

**Authors:** 


Ozena.—In the successful treatment of all forms of fetid catarrh no remedy surpasses Sabalol Spray (J. C. Morgan & Co., N. Y.). It not only promptly overcomes the disagreeable odors that make life a burden for the sufferer, and those who come in contact with him, but also rapidly corrects the underlying conditions.

Rhinologists have found Sabalol Spray an application of broad utility in the successful therapy of nose and throat diseases.

